# Phosphatidylserine exposure promotes increased adhesion in *Dictyostelium* Copine A mutants

**DOI:** 10.1371/journal.pone.0250710

**Published:** 2021-05-27

**Authors:** Amber D. Ide, Elise M. Wight, Cynthia K. Damer

**Affiliations:** Department of Biology, Central Michigan University, Mount Pleasant, Michigan, United States of America; Université de Genève, SWITZERLAND

## Abstract

The phospholipid phosphatidylserine (PS) is a key signaling molecule and binding partner for many intracellular proteins. PS is normally found on the inner surface of the cell membrane, but PS can be flipped to the outer surface in a process called PS exposure. PS exposure is important in many cell functions, yet the mechanisms that control PS exposure have not been extensively studied. Copines (Cpn), found in most eukaryotic organisms, make up a family of calcium-dependent phospholipid binding proteins. In *Dictyostelium*, which has six copine genes, CpnA strongly binds to PS and translocates from the cytosol to the plasma membrane in response to a rise in calcium. Cells lacking the *cpnA* gene (*cpnA*^-^) have defects in adhesion, chemotaxis, membrane trafficking, and cytokinesis. In this study we used both flow cytometry and fluorescent microscopy to show that *cpnA*^-^ cells have increased adhesion to beads and bacteria and that the increased adhesion was not due to changes in the actin cytoskeleton or cell surface proteins. We found that *cpnA*^-^ cells bound higher amounts of Annexin V, a PS binding protein, than parental cells and showed that unlabeled Annexin V reduced the increased cell adhesion property of *cpnA*^-^ cells. We also found that *cpnA*^-^ cells were more sensitive to Polybia-MP1, which binds to external PS and induces cell lysis. Overall, this suggests that *cpnA*^-^ cells have increased PS exposure and this property contributes to the increased cell adhesion of *cpnA*^-^ cells. We conclude that CpnA has a role in the regulation of plasma membrane lipid composition and may act as a negative regulator of PS exposure.

## Introduction

Biological membranes are composed of different types of phospholipids that are not equally distributed between the two leaflets of the lipid bilayer; the outer leaflet of the plasma membrane consists mainly of phosphatidylcholine and sphingomyelin and the inner leaflet consists mainly of phosphatidylethanolamine, phosphatidylserine, and phosphatidylinositol [1]. The phospholipid phosphatidylserine (PS) is a key signaling molecule and binding partner for many intracellular proteins and as such is normally found in the inner leaflet of the plasma membrane. However, PS can be shuttled from the inner to the outer leaflet in a process called PS exposure [1,2].

The asymmetric lipid composition of the plasma membrane is maintained by ATP-dependent flippases that transport PS and PE from the external surface to the cytosolic surface of the plasma membrane [1,3]. Scramblases are calcium-dependent proteins that transport lipids in both directions and play an important role in PS exposure [3]. PS exposure has many functions in animal cells including myoblast fusion, synaptic pruning, fertilization, blood coagulation, and as a phagocytic signal for apoptotic cells [4,5]. PS exposure results in an easily recognizable change to the plasma membrane and serves as a protein binding site to facilitate cell-cell interactions. Scramblases are also found in protist and fungal cells, indicating that PS exposure has ancient origins, and may be important in other fundamental cellular processes [5,6].

Copines are highly conserved, calcium-dependent membrane binding proteins found in many eukaryotic organisms from *Paramecium* to humans [7,8]. Copines contain two C2 domains, which confer calcium-dependent lipid binding activity to proteins, and an A domain, similar to the VWA domain in integrins, that is thought to function as a protein-binding motif [9–11]. The domain structure suggests that in response to a rise in intracellular calcium copines either 1) bind to target proteins and direct them to membranes and/or 2) regulate target proteins at the surface of membranes [11].

There are six copine genes in *Dictyostelium*, *cpnA-cpnF*, that share 28–60% amino acid sequence identity [8]. So far, we have focused our studies on CpnA. We showed that CpnA, like other C2 domain containing proteins, binds strongly to the anionic phospholipids, PS and phosphorylated phosphatidylinositols, in a calcium-dependent manner [12]. In live cells, GFP-tagged CpnA was cytosolic, but translocated from the cytoplasm to the plasma membrane in response to a rise in calcium concentration [12]. We also found that CpnA bound to F-actin in a calcium-dependent manner *in vitro* [13]. Mutant studies showed that *cpnA*^-^ cells had no defects in growth or viability, but did have defects in development [14–16]. *cpnA*^-^ cells also exhibited defects in processes related to membrane trafficking and the actin cytoskeleton including contractile vacuole exocytosis, postlysosome maturation and exocytosis, chemotaxis, and adhesion [13,17]

*Dictyostelium* cells feed on bacteria and yeast by phagocytosis. Phagocytosis requires the attachment of particles to the outside surface of the cell and actin polymerization to remodel the plasma membrane to allow for engulfment of the particles. We began this study by examining the role of CpnA in phagocytosis. We found that *cpnA*^-^ cells have increased adhesion to beads and bacteria. We show that the increased adhesion is not due to changes to membrane proteins or the actin cytoskeleton, but instead due to changes in the lipid composition of the cell membrane. We also found that *cpnA*^-^ cells have increased PS on the external leaflet of the plasma membrane. Overall, we conclude that CpnA has a role in the regulation of plasma membrane lipid composition and may act as a negative regulator of PS exposure.

## Materials and methods

### Cell strains and culture

The parental *Dictyostelium* axenic cell strain, NC4A2 [18], was grown on plastic Petri dishes in HL-5 media (0.75% proteose peptone #2, 0.75% proteose peptone #3, 0.5% yeast extract, 1% glucose, 2.5 mM Na_2_HPO_4_, and 8.8 mM KH_2_PO_4_, pH 6.5) at 18 °C. To prevent bacterial contamination, 60 U/mL of penicillin-streptomycin (Sigma-Aldrich, P4333) was added to the HL-5 media. The *cpnA* knockout cell line, *cpnA*^-^, was created previously by using homologous recombination to replace the *cpnA* gene with the blasticidin resistant gene (*bsr*) and grown in HL-5 containing 10 μg/mL blasticidin (InvivoGen, ant-bl-1) [14]. The bacterial strain, *Klebsiella aerogenes*, expressing GFP was obtained from the Dicty Stock Center and grown in Luria broth (LB) with ampicillin (100 μg/mL) in a shaking suspension culture at 250 rpm for 18 hours at 37 °C [19]. The *cpnA* cDNA was subcloned into the SacI site of the pTX-GFP plasmid for the expression of CpnA with a GFP tag at the N-terminus [12,20]. The plasmid was transformed into NC4A2 and *cpnA*^-^ *Dictyostelium* cells by electroporation and cells were selected with G418 (50 mg/mL). Expression of GFP-tagged CpnA proteins were verified by western blot with an antibody to GFP [12].

### Phagocytosis assays

*Dictyostelium* NC4A2 and *cpnA*^-^ cells were harvested by centrifugation (437 x g, 5 mins, 4 °C) and 2x10^6^ cells/mL were incubated with either GFP-labeled *K*. *aerogenes* at 5x10^7^ cells/mL or 1 μm yellow-green large FluoSphere carboxylate modified microspheres (Thermo-Fisher, F8823) at 3.64x10^10^ beads/mL in 2.5 mL of HL-5 media. Cells were allowed to phagocytose for 30 minutes in a shaking suspension at 180 rpm in a 50 mL flask at room temperature.

For bead phagocytosis, 100 μL of cells were transferred from the flask into a microcentrifuge tube at 5-minute timepoints and cells were washed free of unbound beads by centrifugation at 437 x g for 5 minutes at 4 °C. The cells were resuspended in ice-cold Sorensen’s buffer (0.2 M NaH_2_PO_4_, 0.2 M Na_2_HPO_4_, pH 6.5), washed two times, fixed in 3.7% formaldehyde in Sorensen’s buffer, and again washed two times with Sorensen’s buffer. Cell fluorescence was measured with flow cytometry. To remove beads from the surface of cells before fixation, cells were washed in 5 mM sodium azide (Sigma-Aldrich, 26628-22-8) in Sorensen’s buffer twice and then in Sorensen’s buffer alone. Mean cell fluorescence data from three trials were averaged. Significant differences at each timepoint between NC4A2 and *cpnA*^-^ cells with and without sodium azide washes were analyzed using a repeated measures ANOVA and post hoc Tukey comparisons.

For fluorescence microscopy, NC4A2 and *cpnA*^-^ cells were allowed to phagocytose and washed with and without sodium azide as described above. Cells were plated on glass coverslips and allowed to settle for 15 minutes. Cells were then fixed in cold 3.7% formaldehyde (32% formaldehyde in aqueous solution, Electron Microscopy Sciences, 15714) diluted in methanol for 10 minutes at -20 °C. Fixed cells were washed three times with ice-cold Sorensen’s Buffer with 5 minutes in between washes on coverslips. Coverslips were placed cell side down onto glass slides containing 50% glycerol. Cells were imaged with a Leica DMi 8 microscope. For bacteria phagocytosis, the number of bacteria associated with ~900 individual *Dictyostelium* cells were counted and averaged per trial per sample. Data from the 30-minute timepoint, five trials of buffer alone and three trials of buffer with sodium azide, were averaged and analyzed for significant differences between NC4A2 and *cpnA*^-^ cells using a Student’s t-test.

### Bead adhesion assays

In 50 mL flasks containing 2.5 mL HL-5 media, NC4A2 and *cpnA*^-^ cells were incubated with 0.1 mg/mL Latrunculin A (LatA)(Cayman Chemical, NC0673768) in DMSO or 52.7 μL of DMSO for 30 minutes in a shaking suspension at 180 rpm. After 30 mins, 1 μm beads (3.64x1010 beads/mL) were added to the flasks. After 15 minutes, cells were centrifuged at 437 x g and washed twice with Sorensen’s buffer containing sodium azide or Sorensen’s Buffer alone. Cells were fixed in 3.7% formaldehyde in Sorensen’s Buffer. After fixation and washing cells twice in Sorensen’s buffer, flow cytometry was used to measure mean cell fluorescence. Mean cell fluorescence data for each cell type and condition were normalized to the average mean cell fluorescence within each trial. Normalized data from four trials were averaged and analyzed for significant differences using a Student’s t-test. For fluorescence microscopy, NC4A2 and *cpnA*^-^ cells were allowed to adhere to beads for 30 minutes as described above and then placed on coverslips and fixed in 3.7% formaldehyde in methanol and imaged with a Leica DMi 8 microscope.

For proteinase K bead adhesion assays, NC4A2 and *cpnA*^-^ cells were incubated with LatA for 30 minutes and then 0 μg/mL, 100 μg/mL, and 500 μg/mL of proteinase K for 15 minutes in a shaking suspension. After 15 minutes, 1 μm beads (3.64x1010 beads/mL) were added to the flasks and allowed to adhere to the surface of the cells for 15 minutes at 180 rpm. Cell samples (100 μL) were washed three times with Sorensen’s buffer by centrifugation at 437 x g for 5 minutes at 4 °C. Cells were fixed in 3.7% formaldehyde in Sorensen’s buffer, washed twice in Sorensen’s buffer, and analyzed by flow cytometry. Mean cell fluorescence data were normalized to the average mean cell fluorescence for all cell types and conditions within each trial. Normalized data from three trials of each cell type were averaged and analyzed for significant differences between parental and *cpnA*^-^ cells using an ANOVA and post hoc Tukey comparisons.

Cell samples treated with proteinase K were also analyzed by western blot. Cells were incubated with or without 500 μg/mL proteinase K for 30 minutes in a shaking suspension, washed three times with Sorensen’s buffer, and counted using a hemocytometer for equivalent sample loading. Cells (3x10^6^ cells/mL) were resuspended in 20 μL of 4X sample buffer with 2 mM Phenylmethylsulfonyl fluoride (PMSF) (VWR, 101079–016) and incubated at 95°C for 2 minutes. For Western blotting, a 10% polyacrylamide gel with whole cell protein samples was transferred to a polyvinylidene fluoride (PVDF) membrane. The membrane was cut in half and incubated with blocking buffer (5% dry milk, 0.5% Tween-20 in PBS) for 30 minutes at room temperature. The top half of the membrane was incubated with rabbit polyclonal anti-SibA antibody (1:1000)(Geneva Antibody Facility) [21] and the bottom half was incubated with mouse anti-actin antibody (1:2000) (Santa Cruz, SC4778) in blocking buffer overnight at 4°C. The membranes were washed in 0.5% Tween-20 in PBS and the top membrane was incubated with anti-rabbit HRP-conjugated antibody (1:15000) in blocking buffer and the bottom membrane was incubated with anti-mouse HRP-conjugated antibody(1:15000) for 2 hr at room temperature. The membranes were washed with 0.5% Tween in PBS three times and a chemiluminescence kit (Michigan Diagnostics, PWPD02-16) and BioRad imaging system was used to image the blots.

### Annexin-V assays

NC4A2 and *cpnA*^-^ cells were centrifuged at 437 x g for 5 minutes at 4°C and then resuspended to 1x10^5^ cells/mL in 1X binding buffer (0.01 M HEPES, 0.14 M NaCl, 2.5 mM CaCl_2_). For a positive control, 3 μM of calcium ionophore (Sigma-Aldrich, C7522) was added to 0.5 mL NC4A2 and *cpnA*^-^ cells and allowed to incubate at room temperature for 10 minutes. One drop of Annexin V-APC (Invitrogen, R37176) was added to 0.5 mL of cells treated with ionophore or buffer alone and allowed to incubate for 15 minutes. Live cells were analyzed via flow cytometry.

In a 25 mL flask, 2x10^6^ cells/mL NC4A2 and *cpnA*^-^ cells were incubated with 0.1 mg/mL LatA for 30 minutes in a shaking suspension at 180 rpm. Unlabeled Annexin-V (0.1 μg/mL, BD Pharmagen, 556416) or BSA (0.1 μg/mL) was added to each flask with 1X binding buffer. After 15 minutes, 1 μm beads (3.64x1010 beads/mL) were added to the flasks for 15 minutes. Cell samples (100 μL) were removed from the flasks and centrifuged at 437 x g for 5 minutes at 4 °C. Samples were washed three times with 1X binding buffer and fixed in 3.7% formaldehyde in 1X binding buffer. Fixed cells were washed twice with 1X binding buffer and analyzed by flow cytometry. Mean cell fluorescence was normalized to the average fluorescence of all samples within each trial. Normalized data from three trials was analyzed for significant differences using an ANOVA and post hoc Tukey comparisons.

### Polybia-MP1 cell death assay

In 25 mL flasks containing 1 mL of HL-5 media, 2x10^6^ cells/mL of NC4A2 and *cpnA*^-^ cells, and the same cells expressing GFP-CpnA were incubated with different concentrations (0–5 μM) of Polybia-MP1 (BACHEM, 4099795) for 30 minutes in a shaking suspension at 180 rpm. After 30 minutes, three 100μL cell samples for each cell type and concentration were taken out of the flasks and cells were counted using a hemocytometer. Cell counts at each concentration of Polybia-MP1 (1–5 μM) were normalized to the cell counts for the 0 μM Polybia-MP1 sample for each cell type in each trial. Normalized data from three trials for each cell type were averaged and analyzed for significant differences using an ANOVA and post hoc Tukey comparisons. For Differential Interference Contrast microscopy, cells were plated on glass bottom dishes and allowed to adhere for 15 minutes in HL-5 media. After 15 minutes, the media was replaced with 4 μM Polybia-MP1 in HL-5 media and images were taken every 5 minutes for 15 minutes.

### Flow cytometry

Cell samples were analyzed on a Beckman Coulter CytoFlex Flow Cytometer with the 488 nm laser and FITC detector (525/40) for beads, and with the 638 nm laser and APC detector (660/20) for Annexin V-APC. Forward and side scatter were used to gate for cells and beads. Gated cell events were analyzed for mean cell fluorescence and 10,000 gated events were recorded for each sample.

### Statistical analysis

A repeated measures ANOVA with post hoc Tukey comparisons (R version 3.6.1) was used to analyze significant mean differences for cell populations sampled over time. An ANOVA and post hoc Tukey comparisons (R version 3.6.1) was used to analyze significant differences for cell populations with multiple treatments. A two-sample assuming unequal variances one-tailed t-test (Excel version 16.43) was used to analyze significant mean differences between two cell populations and/or treatments. * indicates p-value<0.05, ns indicates no significant difference, and error bars represent standard error of the mean.

## Results

### *cpnA*^-^ cells have increased adhesion to both beads and bacteria

**Fig 1:**
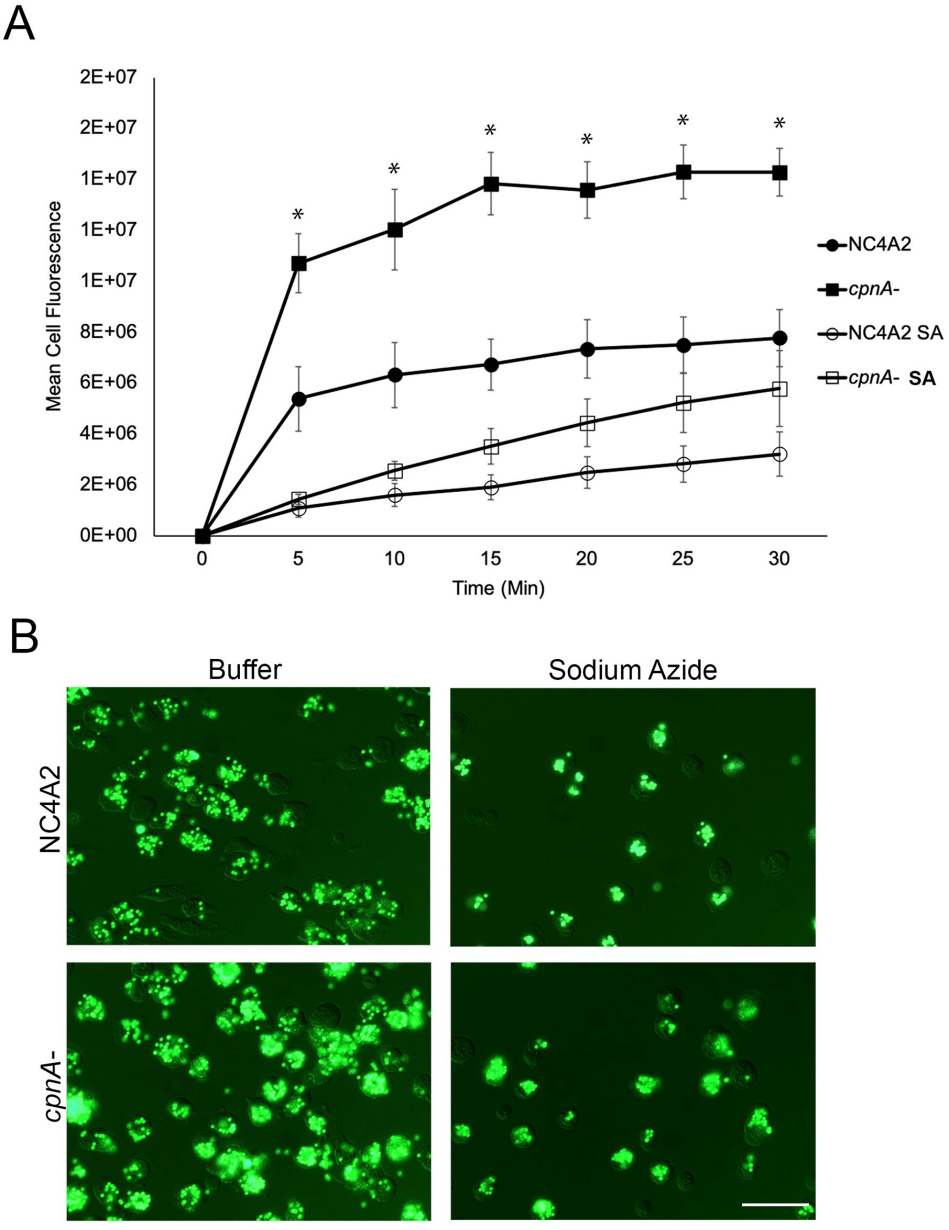
*cpnA*^-^ cells exhibit increased adhesion of 1 μm beads. (A) NC4A2 (squares) and *cpnA*^-^ (circles) cells were incubated with beads. Cell samples were collected at different timepoints, washed in buffer with (open symbols) or without sodium azide (SA) (closed symbols), then fixed and analyzed using flow cytometry. Data from 3 trials (n = 10,000 cells per sample) were analyzed for significant differences using a repeated measures ANOVA and post hoc Tukey comparisons. * indicates significant difference between NC4A2 and *cpnA*^-^ cells without sodium azide, p<0.05. (B) NC4A2 and *cpnA*^-^ cells were incubated with beads, washed in buffer with or without sodium azide, fixed on coverslips, and imaged using DIC and fluorescence microscopy. Scale bar = 25 μm.

To determine if cells lacking the *cpnA* gene exhibited any defects in phagocytosis, we performed phagocytosis assays with both the parental NC4A2 cells and a previously made *cpnA*^-^ cell line. Cells were incubated with 1 μm fluorescent beads over a 30-minute time period and cell samples were analyzed by flow cytometry at 5-minute intervals (Fig 1). *cpnA*^-^ cells had significantly more bead fluorescence compared to the parental cells at all timepoints (Fig 1A, solid shapes). However, because *cpnA*^-^ cells are more adherent to surfaces [13], we investigated whether the increased fluorescence in the *cpnA*^-^ cells was due to increased adhesion of beads to the outside surface of the cells rather than increased phagocytosis. To distinguish adhered beads from phagocytosed beads, we washed the cells with buffer containing sodium azide, which releases particles bound to the cell surface [22]. Therefore, we repeated the phagocytosis assays, but this time washed the cells twice with buffer containing 5 mM sodium azide before fixing for flow cytometry. We found that the sodium azide washes drastically reduced the overall mean cell fluorescence for both cell types (Fig 1A, hollow shapes). Although the mean cell fluorescence of *cpnA*^-^ cells was higher than NC4A2 cells after washes with buffer containing sodium azide, the differences at each time point between NC4A2 and *cpnA-* cells were no longer significant. This suggests that the increased mean cell fluorescence of *cpnA*^-^ cells was due to increased bead adhesion and that increased adhesion resulted in the observed, but not significant, increase in phagocytosed beads at the later timepoints (Fig 1A, hollow shapes). We also used florescence microscopy to examine cells that were incubated with beads for 15 minutes and then washed in buffer with or without sodium azide (Fig 1B). The images appeared to corroborate the flow cytometry data in that fewer beads were associated with cells when washed with sodium azide and most of the beads remaining appeared to be inside the cell and not attached on the outside surface. Overall, these data suggests that the increased bead fluorescence of the *cpnA*^-^ cells was due to the increased adhesion properties of the *cpnA*^-^ cells.

**Fig 2:**
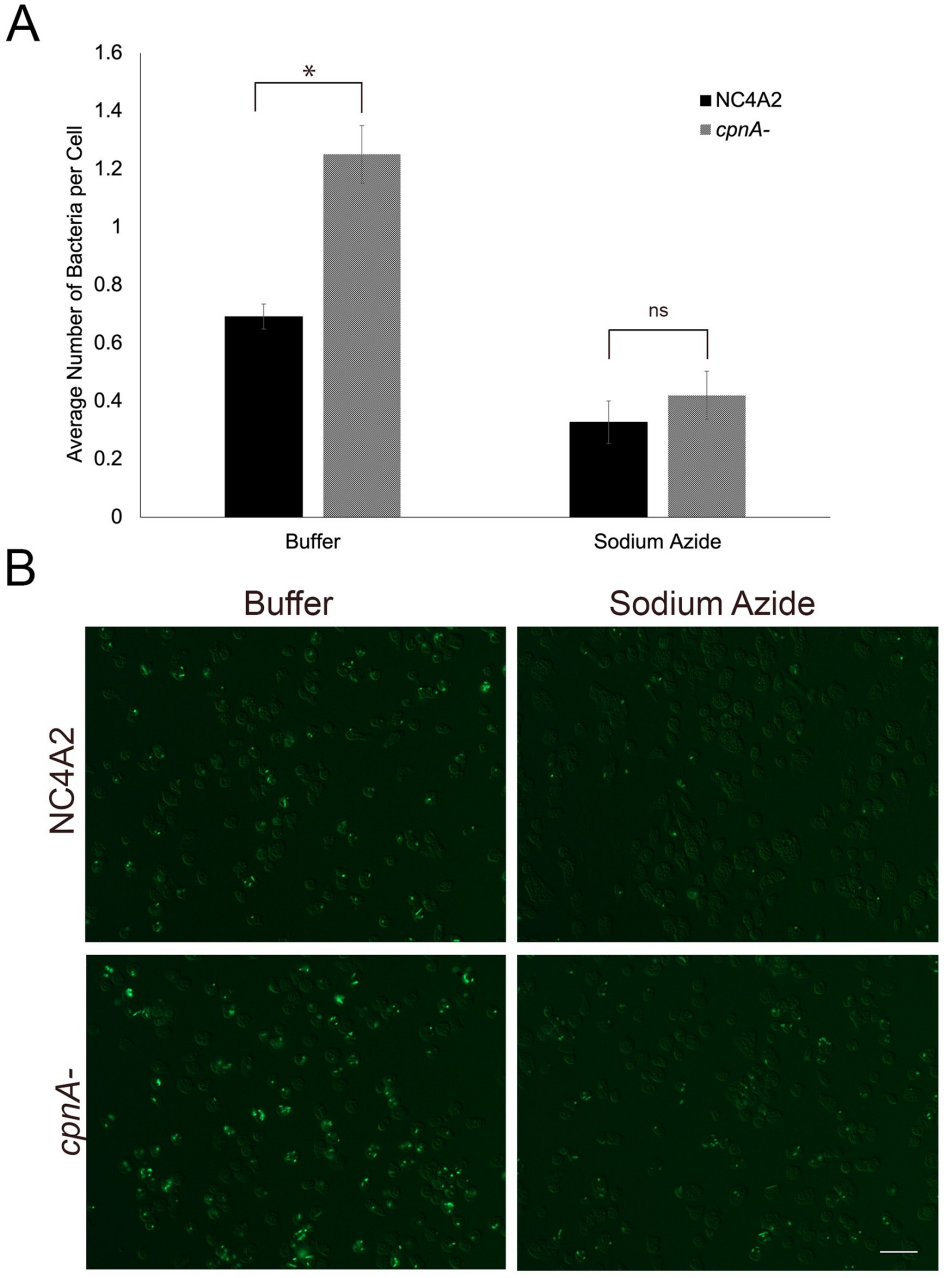
*cpnA*^-^ cells exhibit increased adhesion to GFP-*Klebsiella aerogenes*. NC4A2 and *cpnA*^-^ cells were allowed to phagocytose GFP-bacteria for 30 minutes in a shaking suspension, washed in buffer with or without sodium azide, fixed on coverslips and imaged using DIC and fluorescence microscopy. (A) The number of bacteria associated with each individual cell was counted from the images and the average number of bacteria per cell was calculated. Data from five trials of buffer alone and three trials with sodium azide (n = 900 cells per cell type per trial) were averaged and analyzed for significant differences using a Student’s t-test. * indicates p<0.05 and ns indicates no significant difference. (B) Representative images of fluorescence and DIC microscopy of NC4A2 and *cpnA*^-^ cells with and without sodium azide. Scale bar = 25 μm.

To determine if *cpnA*^-^ cells exhibited increased phagocytosis and/or adhesion to bacteria, we did phagocytosis assays using GFP-expressing *Klebsiella aerogenes* with parental and *cpnA*^-^ cells and imaged cells at the 30-minute timepoint (Fig 2). Cell samples were washed with buffer alone or buffer containing sodium azide and then fixed on coverslips. The cells were imaged with fluorescence microscopy and the number of bacteria per cell were counted (Fig 2). Similar to the results of the bead assay, *cpnA*^-^ cells were associated with significantly more GFP-bacteria compared to parental cells when not washed with buffer containing sodium azide. Yet, when cells were washed with buffer containing sodium azide to rid the cells of surface bound GFP-bacteria, there was no significant difference in the number of associated bacteria per cell. The number of associated bacteria per cell is low due to the GFP being readily quenched once the bacterium is phagocytosed.

### Increased adhesion of *cpnA*^-^ cells is not dependent on actin filaments

**Fig 3:**
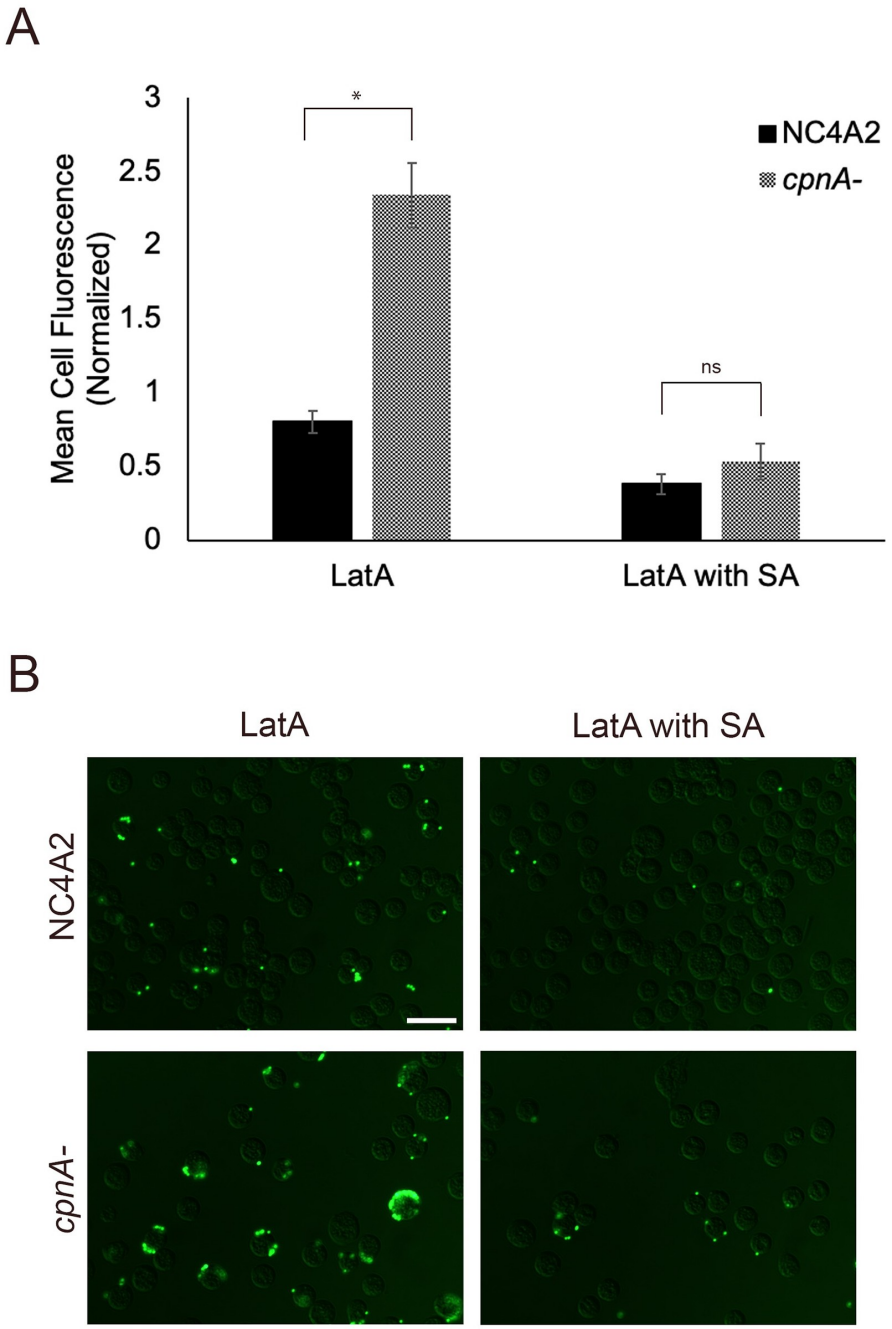
Increased adherence of *cpnA*^-^ cells is not due to increased actin filaments. (A) NC4A2 and *cpnA*^-^ were treated with or without LatA for 30 minutes in a shaking suspension and then incubated with 1 μm beads for 15 minutes. Cells were washed in buffer with or without sodium azide (SA), fixed, and analyzed using flow cytometry. Mean cell fluorescence for each cell type and condition was normalized to the average mean cell fluorescence within each trial. Data from 4 trials (n = 10,000 cells per sample) were analyzed for significant differences using a Student’s t-test. * indicates p<0.05 and ns indicates no significant difference. (B) NC4A2 and *cpnA*^-^ were treated with LatA for 30 minutes in a shaking suspension and incubated with 1 μm beads for 15 minutes. Cells were washed in buffer with or without sodium azide (SA), fixed on coverslips, and imaged using DIC and fluorescence microscopy. Scale bar = 16 μm.

Actin filament dynamics are necessary for the phagocytic engulfment of particles and are important in cell adhesion [23]. We previously showed that CpnA is able to interact with actin filaments in a calcium-dependent manner and may play a role in actin depolymerization at the surface of membranes [13,17]. To further understand CpnA’s role in adhesion, we used the actin depolymerizing drug, Latrunculin A (LatA) to both inhibit phagocytosis [24] and to see if actin filaments play a role in the increased adhesion observed in *cpnA*^-^ cells. If F-actin is a major contributing factor to the increased adherence of beads observed in *cpnA*^-^ cells, then we would expect parental NC4A2 and *cpnA*^-^ cells treated with LatA to have similar amounts of bead adherence. Cells were incubated with LatA for 30 minutes and then incubated with 1 μm beads for 15 additional minutes in a shaking suspension. Cells were washed in buffer alone or containing sodium azide, fixed, and analyzed by flow cytometry (Fig 3A). Because the LatA treatment inhibits phagocytosis, we assume we are only measuring beads adhered to the cell surface. With the LatA treatment, *cpnA*^-^ cells had significantly more fluorescence compared to the parental cells. With both the LatA treatment and sodium azide washes, there were still some beads associated with cells, but there was not a significant difference between parental and *cpnA*^-^ cells (Fig 3A). Images of fixed cells corroborated the flow cytometry data and indicated that the beads were found mainly associated with the surface of cells when treated with LatA and that the sodium azide washes removed most, but not all beads (Fig 3B). If the increased adhesion observed in *cpnA*^-^ cells was solely due to an actin-based adhesion process, we would expect parental and *cpnA*^-^ cells to exhibit similar bead adherence in the absence of actin filaments. Given that *cpnA*^-^ cells exhibited significantly more bead adherence even with LatA treatment, the increased adhesion properties of *cpnA*^-^ cells does not appear to be due to a difference in actin filament dynamics.

### Increased adhesion of *cpnA*^-^ cells is not dependent on cell surface proteins

**Fig 4:**
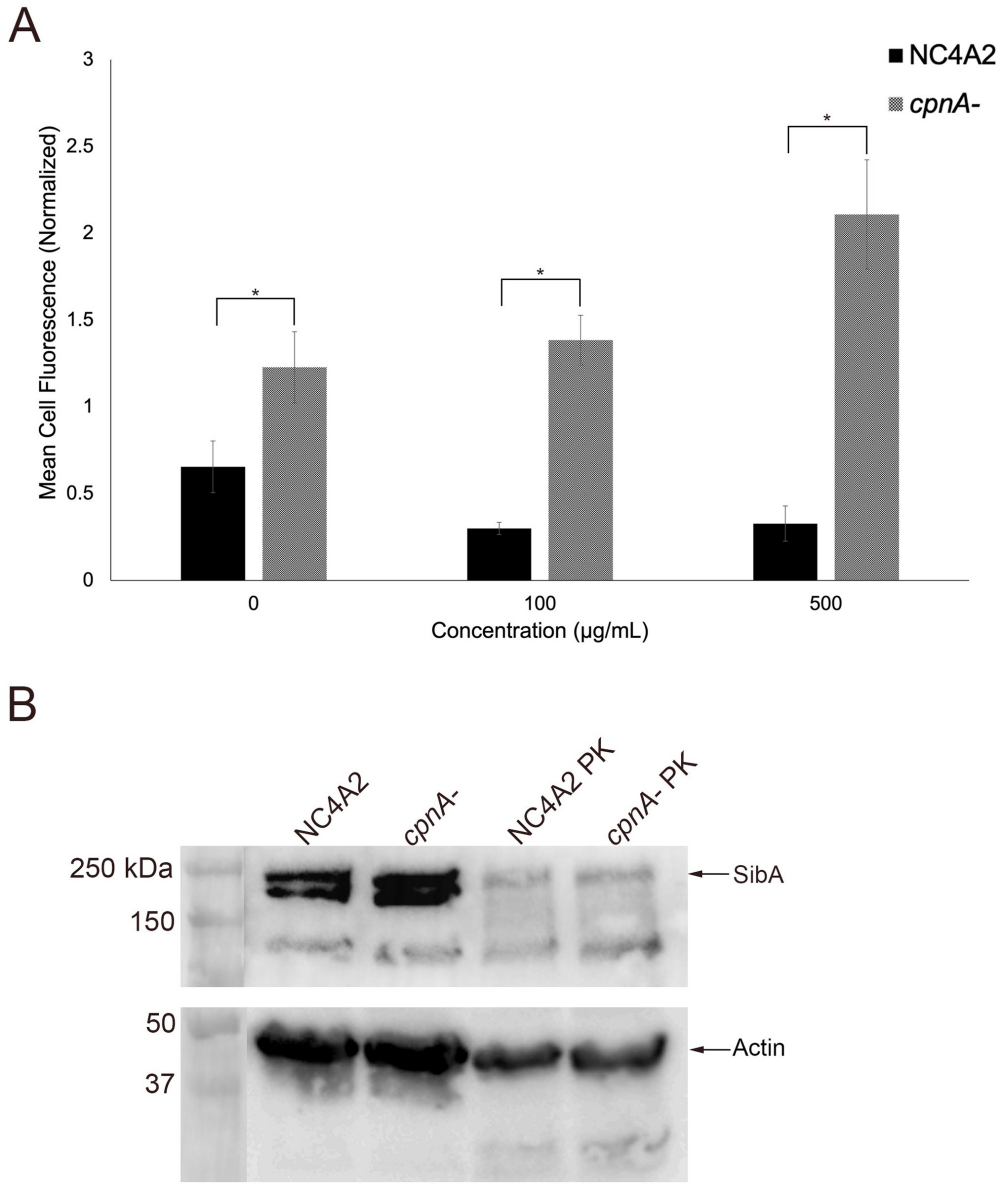
Increased adherence of *cpnA*^-^ cells is not due to cell surface proteins. (A) NC4A2 cells and *cpnA*^-^ cells were incubated with LatA for 30 minutes in a shaking suspension and incubated with different concentrations of proteinase K for 15 minutes. Beads were allowed to adhere to the cells for an additional 15 minutes. Cell samples were washed in buffer, fixed, and analyzed using flow cytometry. Mean cell fluorescence was normalized to the average fluorescence within each trial. Data from 3 trials (n = 10,000 cells per sample) were analyzed for significant differences using an ANOVA and post hoc Tukey comparisons, * indicates p<0.05. (B) NC4A2 and *cpnA*^-^ cells were incubated with proteinase K (PK) for 30 minutes in a shaking suspension. Cell samples (3x10^6^ cells/lane) were analyzed by western blot using a rabbit polyclonal anti-SibA antibody or mouse anti-actin antibody.

The increased cell adhesion to beads could be due to an increase or change in adhesion proteins at the cell surface of *cpnA*^-^ cells. To test this hypothesis, parental and *cpnA*^-^ cells were treated with proteinase K to degrade cell surface proteins before bead adherence was measured. If the increased adhesion was due to proteins on the cell surface, we would expect a decrease in bead adherence of *cpnA*^-^ cells to the level of parental NC4A2 cells when cells were treated with proteinase K. Parental and *cpnA*^-^ cells were treated with LatA first and then incubated with different concentrations of proteinase K for 15 minutes before adding 1 μm beads. The cells were allowed to adhere to beads for 15 minutes in a shaking suspension before samples were washed with buffer and fixed for flow cytometry. Treating the parental cells with 100 μg/mL and 500 μg/mL proteinase K concentrations resulted in a decrease in adhesion of beads (Fig 4A). However, treating *cpnA*^-^ cells with 100 μg/mL of proteinase K did not result in a decrease in adhesion and treating cells with 500 μg/mL resulted in an increase in bead adhesion (Fig 4A). At each proteinase K concentration, the *cpnA*^-^ cells had significantly increased bead adherence compared to NC4A2 cells. These data indicate that proteins on the cell surface of *cpnA*^-^ cells do not contribute to the increased adhesion of beads and suggests that instead of changes in protein composition, changes in the lipid composition of the plasma membrane may be the cause of the increased adhesion.

Although surface proteins may be involved in bead binding, indicated by a decrease in beads adhesion observed with NC4A2 cells, the removal of surface proteins from the *cpnA*^-^ cells may lead to increased bead binding to surface lipids.

To confirm that proteinase K degraded cell surface proteins, whole cell samples were analyzed by a western blot using an antibody to the cell surface protein, SibA (Fig 4B). SibA is a 208 kDa integral membrane protein with a large extracellular domain that is recognized by the antibody used in the western blot. The first two lanes contain whole cell samples of cells that were not treated with the proteinase K, while the next two lanes contain whole cell samples of cells treated with proteinase K. Cells were counted again after Sorensen’s buffer washes and the same number of cells was loaded into each lane. Proteinase K treated and untreated cells were imaged using phase contrast microscopy and appeared similar in cell size and shape. Cells treated with proteinase K had less SibA protein indicating that cell surface proteins were degraded (Fig 4B). As a loading control we also used an antibody to actin on the same blot to verify equivalent sample loading. Similar actin bands were observed when comparing the parental cells to *cpnA*^-^ cells; however, the proteinase K treated cells had slightly less actin. During cell lysis with sample buffer, it appeared some proteinase K was still active. Although we added proteinase inhibitor to the sample buffer, proteinase K is active at high temperatures and in SDS, so it was difficult to completely inhibit.

### *cpnA*^-^ cells have more phosphatidylserine in the outer leaflet of the plasma membrane

**Fig 5:**
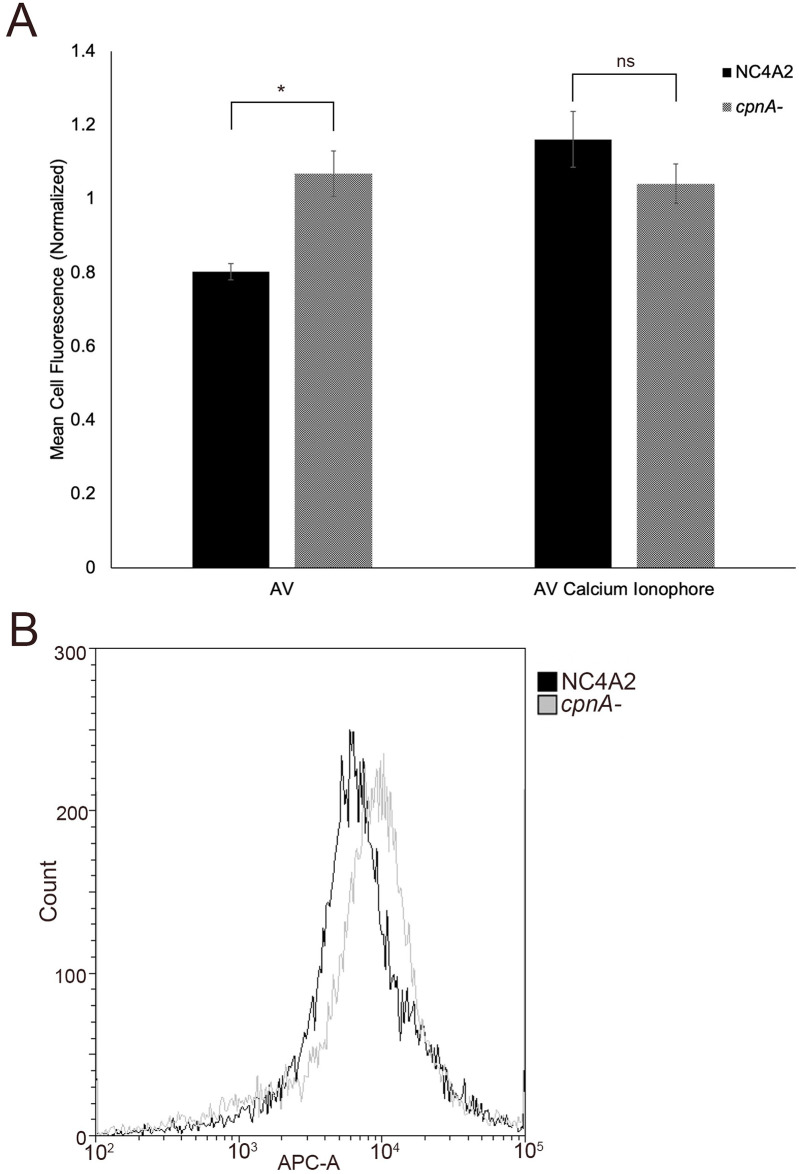
*cpnA*^-^ cells have more PS in the outer leaflet of the plasma membrane. (A) NC4A2 and *cpnA*^-^ cells were incubated in either Annexin V-APC (AV) or calcium ionophore and Annexin V-APC. Cell samples were analyzed using flow cytometry and mean cell fluorescence was normalized to the average mean cell fluorescence for all cell types and conditions within each trial. Data from six trials of Annexin V-APC alone and data from three trials of Annexin V-APC and calcium ionophore (n = 10,000 cells per sample) were analyzed for significant differences using an ANOVA and post hoc Tukey comparisons. * indicates p<0.05 and ns indicates no significant difference. (B) Histogram showing Annexin V-APC fluorescence shift between NC4A2 and *cpnA*^-^ cells.

Because proteins did not appear to be involved in the increased adherence of *cpnA*^-^ cells, we turned to the other main component of the plasma membrane: lipids. The cell membrane is composed of two leaflets, with the outer leaflet composed mainly of phosphatidylcholine and sphingomyelin and the inner leaflet composed of phosphatidylethanolamine, phosphatidylinositol, and phosphatidylserine [1]. We hypothesized that the increased adhesion may be due to a change in lipid composition at the cell surface. To investigate lipids on the cell surface we looked specifically at phosphatidylserine (PS). CpnA binds to PS in both a calcium-dependent and calcium-independent manner [8,12]. We hypothesized that changes to the cell membrane lipid composition could be caused by altered activity of either flippases or scamblases. Both scenarios could lead to *cpnA*^-^ cells having more PS in the outer leaflet of the plasma membrane. To test whether *cpnA*^-^ cells have increased PS in the outer leaflet of the plasma membrane, we used Annexin V-APC to label PS on the surface of NC4A2 and *cpnA*^-^ cells and analyzed cells with flow cytometry (Fig 5). Annexin-V is a calcium-dependent lipid binding protein that specifically binds anionic lipids with a strong affinity for PS. NC4A2 and *cpnA*^-^ cells were incubated in buffer with Annexin V-APC for 15 minutes or with buffer containing a calcium ionophore for 10 minutes, and then incubated with Annexin V-APC for 15 minutes. The cells were immediately analyzed via flow cytometry. We found that *cpnA*^-^ cells had significantly more Annexin V-APC fluorescence compared to the parental cells, indicating that *cpnA*^-^ cells had more PS on the outside of the cells (Fig 5A). This increase in PS exposure can also be observed by a small shift in fluorescence between NC4A2 and *cpnA*^-^ cells (Fig 5B). Stimulation of the cells with a calcium ionophore leads to a rapid elevation of intracellular calcium concentration and subsequent externalization of PS due to the activation of scramblase [25]. We found that *cpnA*^-^ cells had a similar amount of fluorescence as compared to the calcium ionophore treated parental cells (Fig 5A).

### Annexin V binding to *cpnA*^-^ cells decreases adhesion

**Fig 6:**
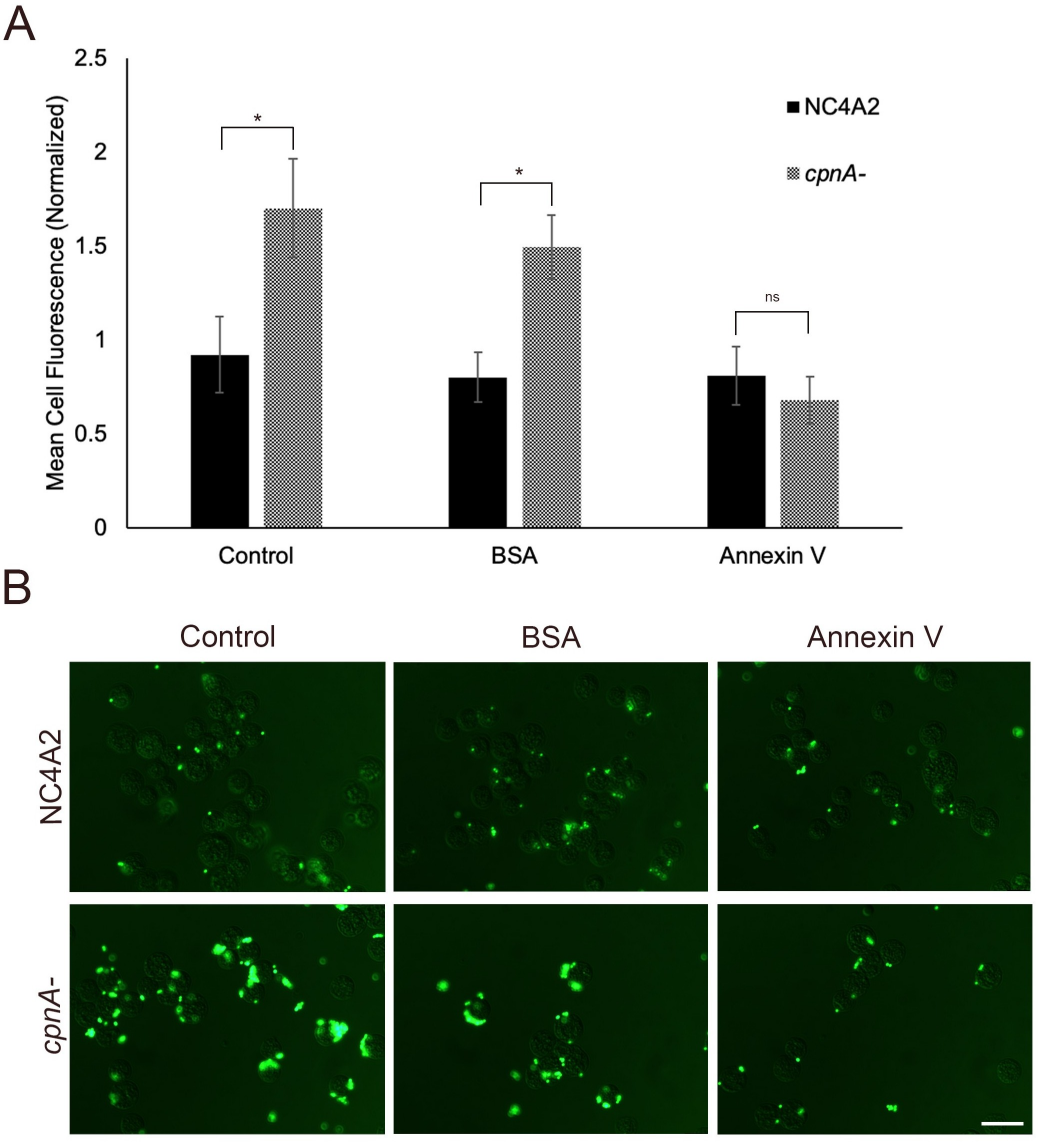
Annexin V blocks increased bead adhesion to *cpnA*^-^ cells. (A) NC4A2 and *cpnA*^-^ cells were incubated with LatA for 30 minutes in a shaking suspension. Cells were incubated with unlabeled Annexin-V or BSA for 15 minutes and then 1 μm beads were added for an additional 15 minutes. Cell samples were washed in buffer, fixed, and analyzed using flow cytometry. Data from 3 trials (n = 10,000 cells per sample) were analyzed for significant differences using an ANOVA and post hoc Tukey comparisons. * indicates p<0.05 and ns indicates no significant difference. (B) NC4A2 and *cpnA-* cells were prepared as above, fixed on coverslips, and imaged using DIC and fluorescence microscopy. Scale bar = 16 μm.

To examine the hypothesis that the increased PS in the outer leaflet of the plasma membrane in *cpnA*^-^ cells contributes to the observed increased adhesion, we tested whether unlabeled Annexin-V could block bead adhesion. Parental and *cpnA*^-^ cells were incubated with LatA in a shaking suspension for 30 minutes and then incubated with either 0.1 μg/mL of Annexin-V or BSA for an additional 15 minutes. We then added 1 μM beads for 15 minutes and fixed cells for flow cytometry. We found that the Annexin V was able to block bead adhesion to the *cpnA*^-^ cells with little effect on the parental cells (Fig 6A). We also used BSA as a control protein for nonspecific protein inhibition of bead adherence. BSA had little effect on bead adhesion in both cell types. Furthermore, NC4A2 and *cpnA*^-^ cells were treated as described above and fixed for fluorescence microscopy (Fig 6B). The fluorescence images also showed that Annexin-V was able to block the increased bead adhesion of *cpnA*^-^ cells. These data suggest that the increased adhesion observed in *cpnA*^-^ cells is likely due to changes to the lipid composition of the plasma membrane and may be specifically due to the increased PS exposure on the plasma membrane.

### *cpnA*^-^ cells are more sensitive to the lytic peptide, Polybia MP-1

**Fig 7:**
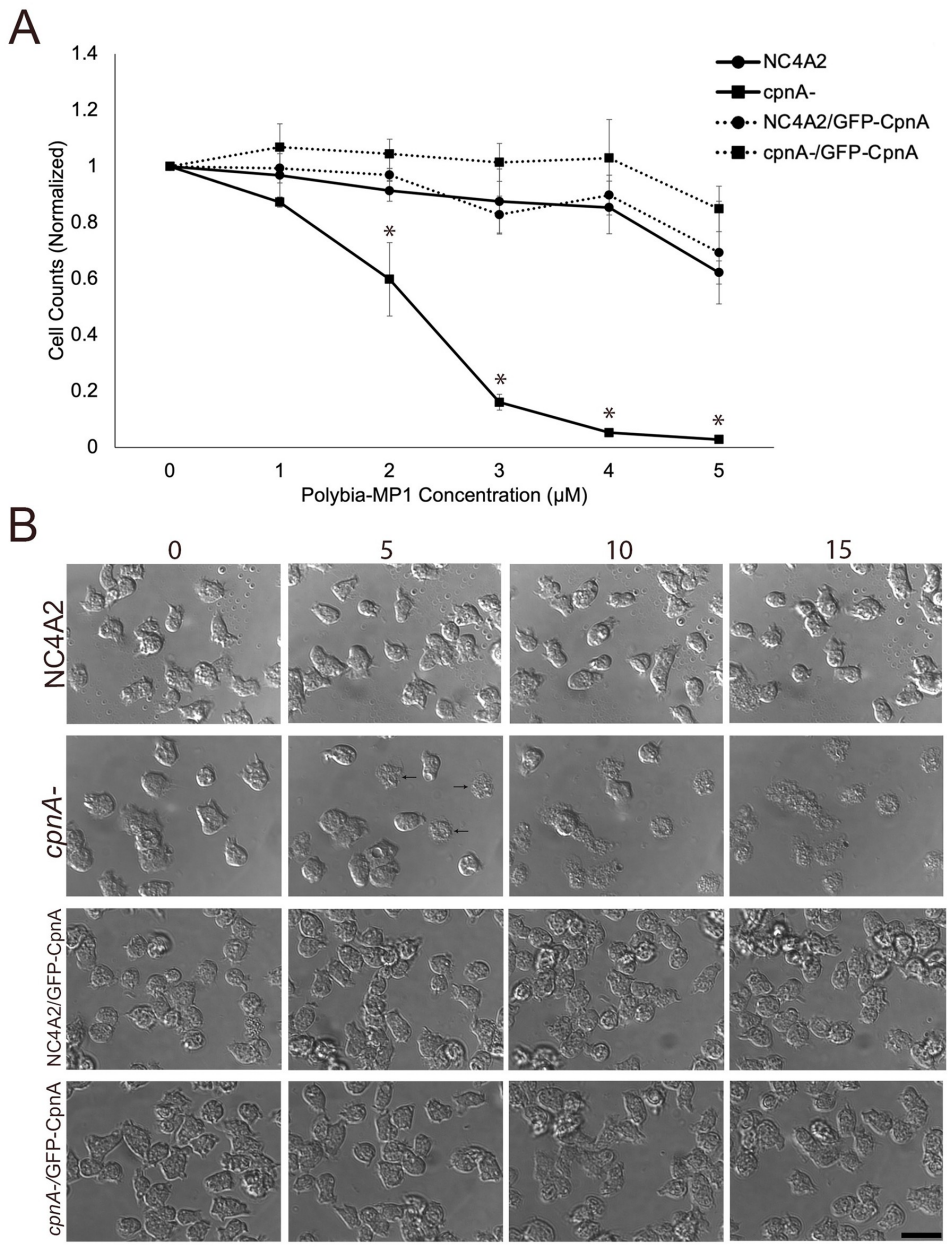
*cpnA*^-^ cells are sensitive to Polybia-MP1. (A) NC4A2 and *cpnA*^-^ cells, and NC4A2 (NC4A2/GFP-CpnA) and *cpnA*^-^ (*cpnA*^-^/GFP-CpnA) overexpressing GFP-tagged CpnA (2x10^6^ cells) were incubated with different concentrations of Polybia-MP1 for 30 minutes in a shaking suspension. Cell samples were counted using a hemocytometer. Cell counts were normalized to 0 μM Polybia-MP1. Data from 3 trials were analyzed for significant differences using an ANOVA and post hoc Tukey comparisons. * indicates significant difference between *cpnA*^-^ and other three cell lines, p<0.05. (B) Cells were plated on glass bottom dishes and treated with 4 μM Polybia-MP1. DIC images were taken every 5 minutes for 15 minutes. Scale bar = 16 μm. Arrows indicate lysed cells.

Another way to determine if *cpnA*^-^ cells have altered plasma membrane lipid composition and increased PS exposure is to test the sensitivity of cells to the lytic peptide found in wasp venom, Polybia-MP1. This peptide binds to external PS residues on the membrane and creates pores in the membrane to induce cell lysis [26]. We placed NC4A2 and *cpnA*^-^ cells in suspension with different concentrations of Polybia-MP1 for 30 minutes and then counted the number of intact cells using a hemocytometer (Fig 7A). For the parental cells, increasing the Polybia-MP1 concentration did not induce cell lysis until 5 μM Polybia-MP1 concentration. However, there was significant cell death of the *cpnA*^-^ cells compared to the parental cells starting at 2 μM Polybia-MP1 and very little cell viability at 4 μM and 5 μM. To see if the overexpression of CpnA could rescue the sensitivity to Polybia-MP1, we overexpressed a GFP-tagged version of CpnA in both the parental and *cpnA*^-^ cell lines. Similar to the parental cell line, both of these cell lines showed very little cell death until 5 μM Polybia-MP1, indicating that expression of CpnA in the *cpnA*^-^ cell line is able to rescue this defect. We also imaged cells after Polybia-MP1 treatment using DIC microscopy. Cells were placed on glass bottom dishes with 4 μM Polybia-MP1 and imaged for 15 minutes (Fig 7B). At 5 minutes *cpnA*^-^ cells started to lyse, while relatively little lysis was observed in the three other cell lines. These data show that *cpnA*^-^ cells are more sensitive to Polybia-MP1, indicating that *cpnA*^-^ cells have more PS exposed on the cell surface than the parental cells.

## Discussion

We began this study by investigating the role of CpnA in phagocytosis and found that more beads and bacteria were associated with *cpnA*^-^ cells than parental cells. Further investigations indicated that *cpnA*^-^ and parental cells had similar amounts of beads and bacteria phagocytosed and that increased adhesion to particles resulted in the increased bead and bacteria association observed with *cpnA*^-^ cells. To investigate how the lack of CpnA may contribute to this increased adhesion, we investigated different cellular components involved in cell adhesion and found that the increased adhesion of *cpnA*^-^ cells was not due to a change in actin filaments at the cell surface nor a change in cell surface proteins. However, we did find that *cpnA*^-^ cells had increased PS in the outer leaflet of the plasma membrane and the increased PS was linked to the increased adhesion.

This study provides the first evidence of a copine protein playing a role in the regulation of plasma membrane lipid composition and possibly PS exposure. In general, the copine family is made up of soluble, cytosolic proteins with calcium-dependent phospholipid binding activity [12]. Copines do not exhibit any homology to other proteins, like flippases and scramblases, that are able to transport PS from one side of a membrane to the other. Therefore, we propose that CpnA does not directly function in phospholipid transport, but has an indirect or regulatory role. Previously, we showed that copines in *Dictyostelium* translocate from the cytosol to the plasma membrane in response to a rise in calcium concentration [12]. Once at the plasma membrane, copines could interact with and regulate plasma membrane proteins that are involved in the transport or flipping of lipids.

Most of the processes in animal cells that require PS exposure involve some form of cell-to-cell adhesion. Examples include phagocytic cells recognizing and engulfing apoptotic cells; red blood cell and platelet aggregation; sperm and egg fertilization, and myocyte fusion [5]. Our results also suggest a role for PS exposure in cell-to-cell adhesion in that *cpnA*^-^ cells have increased adhesion to bacterial cells. In addition, our previous studies showed that *cpnA*^-^ cells have development defects indicative of increased cell-to-cell adhesion [15,16]. *Dictyostelium* can live as single-celled amoebae. However, when placed in starvation conditions, cells undergo chemotaxis in response to secreted cAMP and aggregate into mounds [27]. The mound elongates to form a finger-shaped structure that falls over and migrates as a slug before culminating into a fruiting body consisting of a stalk, made up of cells that have undergone programmed cell death, with a mass of spores on top [27]. We found that *cpnA*^-^ cells formed larger than normal mounds and slugs did not culminate into fruiting bodies [14–16]. When *cpnA*^-^ cells were mixed with increasing percentages of parental cells, mixed cell populations were able to make progressively more normal slugs and fruiting bodies [15,16]. *cpnA*^-^ cells also formed small aggregates within the mixed population slugs [15]. Interestingly, when *cpnA*^-^ cells were developed in buffer containing EGTA, they were able to complete the developmental cycle and culminate into small fruiting bodies [15].

In light of our new data, we hypothesize that the increased cell adhesion observed during development of *cpnA*^-^ cells may be due to altered cell surface lipid composition. The result that EGTA rescued the developmental culmination defect suggests a role for calcium in the increased adhesion of *cpnA*^-^ cells. The development of *cpnA*^-^ cells in buffer containing EGTA may decrease intracellular calcium levels, resulting in decreased scramblase activity. Alternatively, EGTA may reduce the calcium-dependent binding of other proteins that mediate adhesion to PS. One possibility for how the lack of CpnA causes increased adhesion is that CpnA has a role in the negative regulation of scramblase. Recent evidence indicates that there are two main protein families (XKr8 and TMEM16) involved in scramblase activity at the plasma membrane in animal cells [28]. *Dictyostelium* does not appear to have any XKr8 family members, but has a single member of the TMEM16 family [6]. *Dictyostelium* TMEM16 was shown to confer calcium-dependent scramblase activity when expressed in HEK293 cells [6]. CpnA could directly interact with this scramblase or indirectly regulate the activity of the scramblase by playing a role in the negative regulation of intracellular calcium levels.

In animal cells, PS exposed on the surface of cells acts as binding sites for proteins. For example, the PS exposed on activated platelets serves as assembly sites for tenase and prothrombinase complexes [29]. More specifically, the PS acts a binding site for negatively charged carboxyglutamate (Gla) residues at the N-termini of multiple coagulation factors via Ca^2+^ ions [29]. A similar type of calcium ion bridge may be occurring between the PS exposed on *cpnA*^-^ cells and the carboxylate-modified beads used in our assays.

Altered membrane lipid composition and/or calcium concentrations in *cpnA*^-^ cells may also contribute to the increased postlysosome exocytosis phenotype we previously reported [17]. Other studies have reported a link between PS exposure and membrane trafficking. A study with PC12 cells showed that increasing PS levels increased calcium-triggered exocytosis [30], while a study with Jurkat cells showed that calcium activation of scramblase triggered both PS exposure and membrane expansion [31]. Alternatively, increased exocytosis may be responsible for the increased PS on the cell surface of *cpnA*^-^ cells.

Future studies will focus on identifying how CpnA is involved in the negative regulation of PS exposure and how PS exposure is involved in *Dictyostelium* development. During development, prestalk cells lose lipid asymmetry in that they expose PS in the outer leaflet of the plasma membrane [32] and our previous studies indicate CpnA plays a role in the differentiation of prestalk cells [15,16]. Therefore, PS exposure is most likely a tightly regulated process necessary for development and other fundamental cell processes. Cancer cells also have increased PS exposure and this is thought to be caused by low flippase activity and/or high scramblase activity [33,34]. Therefore, uncovering key regulators of PS exposure and the link to adhesion will be important to our understanding of tumorigenesis and cancer metastasis.
